# MED30 Regulates the Proliferation and Motility of Gastric Cancer Cells

**DOI:** 10.1371/journal.pone.0130826

**Published:** 2015-06-25

**Authors:** Yong Joo Lee, Myoung-Eun Han, Su-Jin Baek, Seon-Young Kim, Sae-Ock Oh

**Affiliations:** 1 Department of Anatomy, School of Medicine, Pusan National University, Busan, Republic of Korea; 2 Medical Research Center for Ischemic Tissue Regeneration, Pusan National University, Busan, Republic of Korea; 3 Medical Genomics Research Center, KRIBB, Daejeon, Republic of Korea; Wayne State University School of Medicine, UNITED STATES

## Abstract

MED30 is an essential member of the mediator complex that forms a hub between transcriptional activators and RNA polymerase II. However, the expressions and roles of MED30 have been poorly characterized in cancer. In this study, we examined the functional roles of MED30 during gastric cancer progression. It was found that MED30 was overexpressed in gastric cancer tissues and cell lines. Moreover, MED30 overexpression increased the proliferation, migration, and invasion of gastric cancer cells, whereas MED30 knockdown inhibited these effects. Furthermore the knockdown significantly inhibited tumorigenicity in SCID mice. MED30 also promoted the expressions of genes related to epithelial-mesenchymal transition and induced a fibroblast-like morphology. This study shows MED30 has pathophysiological roles in the proliferation, migration, and invasion of gastric cancer cells and suggests that MED30 should be viewed as a potent therapeutic target for malignant gastric carcinoma

## Introduction

Gastric cancer is one of the leading causes of cancer death worldwide [1, 2]. Approximately 95% of gastric cancers are adenocarcinomas and in epidemiological studies gastric cancer has been classified by anatomic site as cardia/proximal or noncardia/distal [3] and by histological phenotype as intestinal, diffuse, or mixed/unclassifiable as described by Lauren [4]. Furthermore, patients with proximal gastric cancer have poorer survival independent of TNM stage [5]. *Helicobacter pylori* infection has been demonstrated to be an etiologic agent of gastric cancer, particularly of cancers found in the distal stomachs of elderly males, which are usually of the intestinal type. More recently, several molecular classifications of gastric cancer have been proposed based on the findings of whole-genome gene expression studies and/or gene copy number studies [6–10].

Transcriptional regulation is a crucial step that controls cell identity, growth, differentiation, and development. Human mediator (MED) complex, which contains ~30 proteins, is a key coactivator/activator of the expressions of RNA polymerase II (Pol II)-transcribed genes [11]. MED complex facilitates the pre-initiation complex (PIC) assembly by interaction with Pol II and gene specific transcription factors (TFs), such as, TFIIA, TFIIB, TFIID, TFIIE, TFIIF, and TFIIH [12, 13]. MED complex consists of three distinct structural submodules (head, middle, and tail). The head module directly interacts with Pol II, whereas the elongated tail module interacts with gene-specific regulatory proteins [12, 13], and the middle module acts in regulatory signal transfer at a post-binding stage [13]. Although the mechanism is not fully understood, MED complex tightly binds to Pol II, changes its conformation and affects the transcription initiation process [14, 15]. Since MED complex is an essential component of the transcription machinery, most of subunits in the core of MED are required for embryonic growth and cell viability [16].

Cancer genome sequencing studies have reported mutations or alterations in the RNA transcription machinery components contained in MED subunits, and correlations between some of these changes in MED subunits, (MED1, MED12, MED19, MED23, MED28, CDK8, and cyclin C) and cancer progression have been reported for various cancers, although mechanisms responsible for these correlations are unknown [17].

Recently, it was reported that a MED19 can participate in gastric cancer progression, as its knockdown significantly inhibited cell proliferation and colony-formation capacity, and induced G1 phase cell-cycle arrest in two human gastric cancer cell lines (SGC7901 and MGC803) [18]. However, the functional roles and pathological relevance of other MED subunits in gastric cancer remain unclear.

In the present study, to reveal the functional importance of MED30 during gastric cancer progression, we examined its roles in proliferation, migration, invasion and tumorigenicity of gastric cancer cells. Before the functional examination, we checked the expression pattern of MED30 in gastric cancer cells and tissues.

## Materials and Methods

### Cell cultures and transfection

Gastric cancer cell lines (SNU1, SNU16, SNU216, SNU620, SNU638, and N87) were purchased from the Korean Cell Line Bank (Seoul). Cells were cultured in RPMI1640 supplemented with 25 mM HEPES, 10% fetal bovine serum (FBS), and 100 μg/ml of penicillin/streptomycin (1 × P/S) in 5%CO_2_/95% air at 37°C. Cells were transfected with siRNA using DharmaFECT reagent 1 or 3 (Dharmacon, Lafayette, CO), according to the manufacturer’s instructions. The sequences of siRNA used were as follows: MED30 siRNA (Bioneer, Daejeon, Korea), 5’-CGA GCA ACU UAU UCC AUA U(dTdT)-3’, 5’-GCU GCC AAA UGG UGU CAC U(dTdT)-3’, and 5’-CGA GAA AUU GCU GAA GUA A(dTdT)-3’; scrambled (SCR) siRNA (Dharmacon, Lafayette, CO), 5’- GAU CCG CAA AAG AGC GAA A(dTdT)-3’.

### MED30 overexpression

In order to construct MED30-over expression vector, we used pLenti6.3-V5/DEST lentiviral vector (Invitrogen, Carlsbad, CA). Briefly, MED30 cDNA was cloned into pLenti6.3-V5/DEST vector using the *in vivo* recombination-based Gateway cloning system (Invitrogen). The donor vector (pDONR221) containing the coding sequence of MED30 (MED30 cDNA) was purchased from Ultimate^TM^ ORF Clones (Invitrogen), and recombined with the counter-selectable *ccdB* gene of the Gateway destination vector pLenti6.3-V5/DEST using the LR clonase enzyme mixture (Invitrogen). The empty vector pLenti6.3/V5-DEST was used as a mock control. Recombinant lentiviruses were produced in 293FT cells, and used to infect SNU638 cells according to the manufacturer’s instructions (ViraPower Lentiviral Expression System; Invitrogen). Stable cell lines were established by selection with blasticidin (7.5 μg/ml) (Invitrogen).

### Real-time PCR

Gastric cancer tissues were obtained after obtaining written informed consent from patients who underwent surgical resection at Pusan National University Hospital or Pusan National University Yangsan Hospital. The study was approved by the Pusan National University Hospital-Institutional Review Board (PNUH-IRB) and the Pusan National University Yangsan Hospital-Institutional Review Board (PNUYH-IRB). Total RNA was extracted from tissues or cells using Trizol reagent (Invitrogen) or the RNeasy Mini kit (Qiagen, Valencia, CA), according to the manufacturer’s instructions. cDNA was synthesized using MMLV reverse transcriptase (Promega, Madison, WI), dNTP, and oligo-dT primers. The sequences of the primers used were as follows: MED30, 5’- ACC GGT TAA CAA AGC TAC AGG A -3’ (sense) and 5’- TAA GTT GCT CGA CTG GAA TGG G -3’ (antisense); CDH1, 5’- TGG GCC AGG AAA TCA CAT CC -3’ and 5’- CTC AGC CCG AGT GGA AAT GG -3’; CDH2, 5’- CAC TGT GGA GCC TGA TGC CA -3’ and 5’- TCC CCA ATG TCT CCA GGG TG -3’; CDH3, 5’- CCC CCA GAA GTA CGA GGC CC -3’ and 5’- ACG CCA CGC TGG TGA GTT GG -3’; TWIST1, 5’- CGG GAG TCC GCA GTC TTA -3’ and 5’- TGG ATC TTG CTC AGC TTG TC -3’; TWIST2, 5’- CTT ATG TTT GGG GGG AGG TT -3’ and 5’- TAG CCA AGC AAT CAC GGA GA -3’; VIM, 5’- TGA GTA CCG GAG ACA GGT GCA G -3’ and 5’- TAG CAG CTT CAA CGG CAA AGT TC -3’; SNAI1, 5’- GAG GCG GTG GCA GAC TAG -3’ and 5’- GAC ACA TCG GTC AGA CCA G -3’; SNAI2, 5’- TAG GAA GAG ATC TGC CAG AC -3’ and 5’- CCC CAA GGC ACA TAC TGT TA -3’; GAPDH, 5’- GCA GCC TCC CGC TTC GCT CT -3’ and 5’- TGG TGA CCA GGC GCC CAA TAC G -3’. Real-time PCR was performed using a LightCycler^TM^ 96 Real-time PCR system (Roche, Nutley, NJ, USA) with FastStart Essential DNA Green Master (Roche), according to the manufacturer’s instructions. GAPDH was used as the internal control.

### Western blot analysis

Cells were lysed with RIPA buffer, protease inhibitor cocktail was added, and protein concentrations were determined using the Bio-Rad protein assay kit (Bio-Rad, Hercules, CA). Samples (80 μg/well) were subjected to SDS-PAGE and then transferred to PVDF membranes. Anti-MED30 (1:500, Protein Tech #16787-1-AP, Chicago, IL), anti-E-cadherin (1:1000, BD Bioscience #610181, San Jose, CA), anti-N-cadherin (1:1000, BD Bioscience #610920), anti-P-cadherin (1:1000, BD Bioscience #610227), anti-Twist1/2 (1:750, Santa Cruz Biotechnology #sc-15393, Santa Cruz, CA), anti-vimentin (1:1000, DAKO #M7020, Carpentaria, CA) and anti-β-actin (1:2000, Santa Cruz Biotechnology #sc-47778) antibodies were diluted in 5% skim milk and incubated with membranes at 4°C overnight. The appropriate secondary antibody was applied (1:2000, horseradish peroxidase-conjugated anti-mouse or anti-rabbit) at room temperature for 1 hr. Chemiluminescence detection (GE Health Care, Little Chalfont, United Kingdom) was used to visualize MED30, E-cadherin, N-cadherin, P-cadherin, Twist1, vimentin, and β-actin. Western blot analysis was performed in triplicate.

### Immunohistochemistry

Tissue microarray sections containing gastric cancer tissues from patients were obtained from SuperBiochip (Seoul). Histopathologic diagnoses were performed at the Department of Pathology, Pusan National University Hospital, and clinicopathologic staging was performed using the TNM classification (AJCC, 7th ed.). All patients had histologically confirmed gastric cancer, and tumor samples were checked to ensure that tumor tissue composed more than 80% of samples. For immunohistochemistry, slides were deparaffinized and rehydrated, treated with 0.3% hydrogen peroxide for 30 min to quench endogenous peroxidase activity, and blocked with 10% normal donkey serum (NDS) and 1% BSA in 1 × phosphate buffered saline (PBS). Slides were then incubated overnight at 4°C in blocking buffer containing primary antibody (anti-human MED30; 1:50, Proteintech). Secondary antibody (HRP-conjugated) binding was performed at a dilution of 1:200 in blocking buffer for 2 hr at RT. Slides were then stained for HRP (Vector Laboratories) and counterstained with hematoxylin staining buffer (Sigma-Aldrich, St. Louis, MO) for 1 min. Percentages of cells that stained for MED30 in sections were determined and sections were then graded using a 1–4 scale (1, <24%; 2, 25–49%; 3, 50–74%; 4, 75–100%). Intensities of tumor cell staining were graded as 0, 1, 2, or 3, which corresponded to basal, weak, moderate, and strong respectively. Overall staining was then represented by composite scores, which were calculated by multiplying these two grades. Accordingly, overall stained was graded from 0 to 12.

### Cell proliferation assay

To examine the effect of siRNA on the proliferation of gastric cancer cells, culture media were replaced with 1% FBS medium one day after the siRNA transfection. Five days after the transfection, 10 μl of Ez-Cytox (ITSBIO, Seoul) was added and cells were incubated for 0.5 to 2 hr under normal culture conditions. Then the absorbance was measured at 450 nm using an ELISA reader (TECAN, Mannedorf, Switzerland). To examine the effect of MED30 overexpression, cells were seeded in 1% FBS medium. Three days after seeding 10 μl of Ez-Cytox was added.

### Boyden chamber assay

The bottom chamber of a transwell chamber was filled with medium containing 10% FBS. One day after transfection with scrambled (SCR) or MED30 siRNA, gastric cancer cells were seeded into the upper chamber at a density of 5 × 10^5^ cells/ml in 50 μl of serum-free medium. To examine the effects of MED30 overexpression, cells (mock and MED30-over) were seeded at a density 1 × 10^5^ cells/ml. To eliminate proliferation associated effects, mitomycin C (0.01 μg/ml, Sigma-Aldrich) was added. Cells were allowed to migrate for four or six hrs. Membranes were then fixed and stained using Diff-quik solution (Sysmex, Kobe, Japan) and washed with distilled water. Cell numbers in 10 randomly chosen fields were counted using a light microscope.

### Matrigel invasion assay

The ability of gastric cancer cells to invade was investigated using 24-well BioCoat^TM^ Matrigel^TM^ chamber inserts (BD Bioscience, San Jose, CA, USA). The top surface of inserts in invasion chambers were coated with 0.5 mg/ml growth factor-reduced Matrigel (BD Bioscience) and the bottom surface with 0.5 mg/ml of fibronectin (Sigma-Aldrich). One day after transfection with SCR or MED30 siRNA, cells were seeded at a density of 5 × 10^4^ cells/ml in serum-free medium into 8-μm porous BioCoat Matrigel chamber inserts, and placed in wells filled with 750 μl of medium supplemented with 10% FBS as a chemoattractant. To examine the effects of MED30 overexpression, cells (mock and MED30-overexpressing cells) were seeded at a density 1 × 10^4^ cells/ml. To remove proliferation-associated effects, mitomycin C (0.01 μg/ml, Sigma-Aldrich) was added. After incubation for 24 hrs, non-invading cells on top surfaces of membranes were removed by scraping. Invading cells on bottom surfaces were fixed, and stained with Diff-quik solution. Experiments were performed in triplicate, and at least 5 fields were counted per experiment.

### Xenograft assay

The SNU638 cells were transfected with SCR or MED30 siRNA. After 2 days, cells were collected by trypsinization, washed twice with PBS, and were injected subcutaneously (1 × 10^6^ cells in 100 μl PBS) into severe combined immunodeficiency (SCID) mice. Tumor volumes were calculated every week from week three to week seven after the injection using the following formula: tumor volume (mm^3^) = (*a* × *b* ^2^)/2, where *a* = length in mm and *b* = width in mm. At seven weeks, mice were sacrificed and tumor volumes and weights were recorded. This experiment was performed in strict accordance with the recommendations in the Guide for the Care and Use of Laboratory Animals issued by the National Institute of Health. The Pusan National University Institutional Animal Care and Use Committee (PNUIACUC) approved the experimental protocol.

### Statistical analysis

Results are presented as means±SDs. The nonparametric Mann-Whitney U-test or the Student’s t-test (unpaired) was used to determine the significances of differences between the mean values of two groups, and one-way analysis of variance (ANOVA) followed by Tukey’s multiple comparisons was used to analyze three or more groups. * indicates a *P* value of <0.05. Survival time was defined as the time elapsed between treatment and death or until the last objective follow-up information was obtained. The Kaplan-Meier method was used to determine the relation between survival and MED30 expression. Curves were compared using the log-rank test at a significance level of 95%. *P* values of <0.05 were considered to be statistically significant. Data were analyzed using SPSS software version 12.0 (SPSS Inc., Chicago, IL, USA).

## Results

### Overexpression of MED30 in gastric cancer tissues

**Fig 1:**
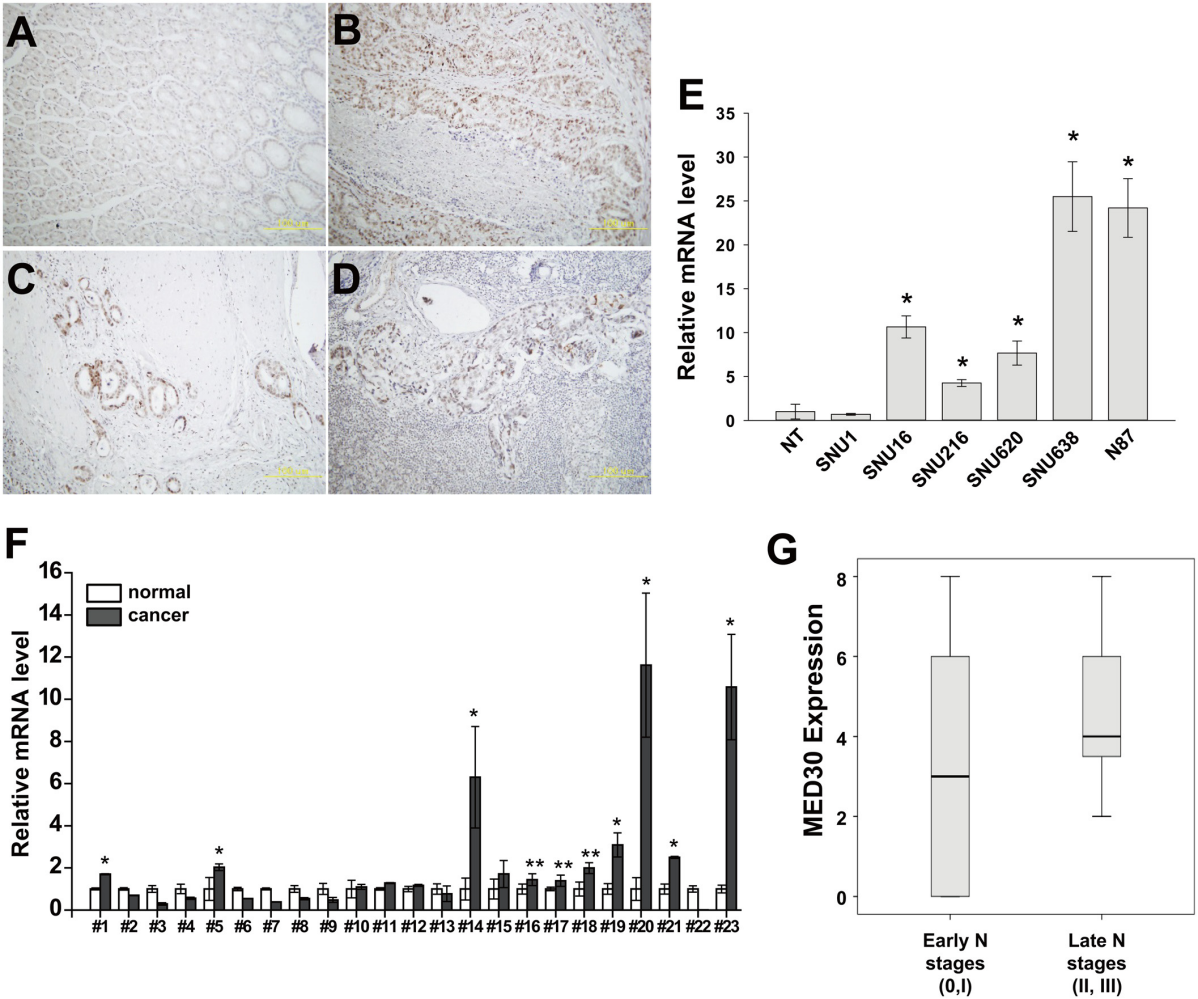
MED30 was overexpressed in gastric cancer tissues. (A-D) Immunohistochemical staining showed the overexpression of MED30 in gastric cancer tissues (B-D) versus normal gastric mucosae (A). Its overexpression was observed in invading cancer cells (C) and metastatic cancer cells nearby lymph nodes (D) in addition to the primary cancer site (B). (E) The overexpression of MED30 in gastric cancer cells was determined by real-time PCR using specific primers (NT; normal tissue). GAPDH was used to normalize all data. Values are the means ± SDs of the three independent experiments performed in triplicate. *, *p*< 0.01 (Student’s *t* test, versus NT). (F) The overexpression of MED30 in gastric cancer tissues was examined by real-time PCR using specific primers. GAPDH was used to normalize all data. Values are the means ± SDs of the three independent experiments performed in triplicate. *, *p*< 0.01; **, *p*< 0.05 (Student’s *t* test, versus normal gastric tissues). (G) MED30 protein expression in higher N stages (N stages 2 and 3) was significantly greater than in lower N stages (N stage 0, 1) (N = 41, *p*< 0.05, Mann-Whitney U test).

To investigate roles played by MED30 in gastric cancer, we first examined the expression status of MED30 in the tumor tissues of 23 gastric cancer patients. MED30 protein was found to be widely overexpressed in cancerous tissue regions (Fig 1A–1D), and was obviously overexpressed in invading gastric cancer cells (Fig 1C) and in metastatic cancer cells inside affected lymph nodes (Fig 1D). In order to validate our immunohistochemistry results, we performed quantitative real-time PCR on gastric cancer tissues using MED30-specific primers. As shown in Fig 1F, DPY30 was overexpressed in 10 cases (10/23, 43%). We also examined the expression pattern of MED30 in gastric cancer cell lines by real-time PCR, and found it to be overexpressed in five of the gastric cancer cell lines tested (except SNU1) versus normal gastric tissues (Fig 1E). To investigate the correlation between MED30 expression and clinical characteristics, we performed immunohistochemistry using 41 gastric cancer tissue samples (Table 1). Interestingly, the expression of MED30 was positively correlated with N stage (Fig 1G) but not with patient survival (data not shown).

**Table 1:** Correlation between the expression of MED30 and clinical classification in the gastric cancer.

		Immunoreactivity Case No (%)		
	Total	Weak	Strong	*P*-value
**Age**				0.146
≤ 60	24	10	14	
60 ≤	17	11	6	
**Gender**				0.623
M	23	11	12	
F	18	10	8	
**N stage**				0.017
N0 ~ N1	26	17	9	
N2 ~ N3	15	4	11	
**TNM stage**				0.082
0 ~ 2	26	16	10	
3 ~ 4	15	5	10	

### Roles of MED30 in the proliferation, migration, and invasion of gastric cancer cells

**Fig 2:**
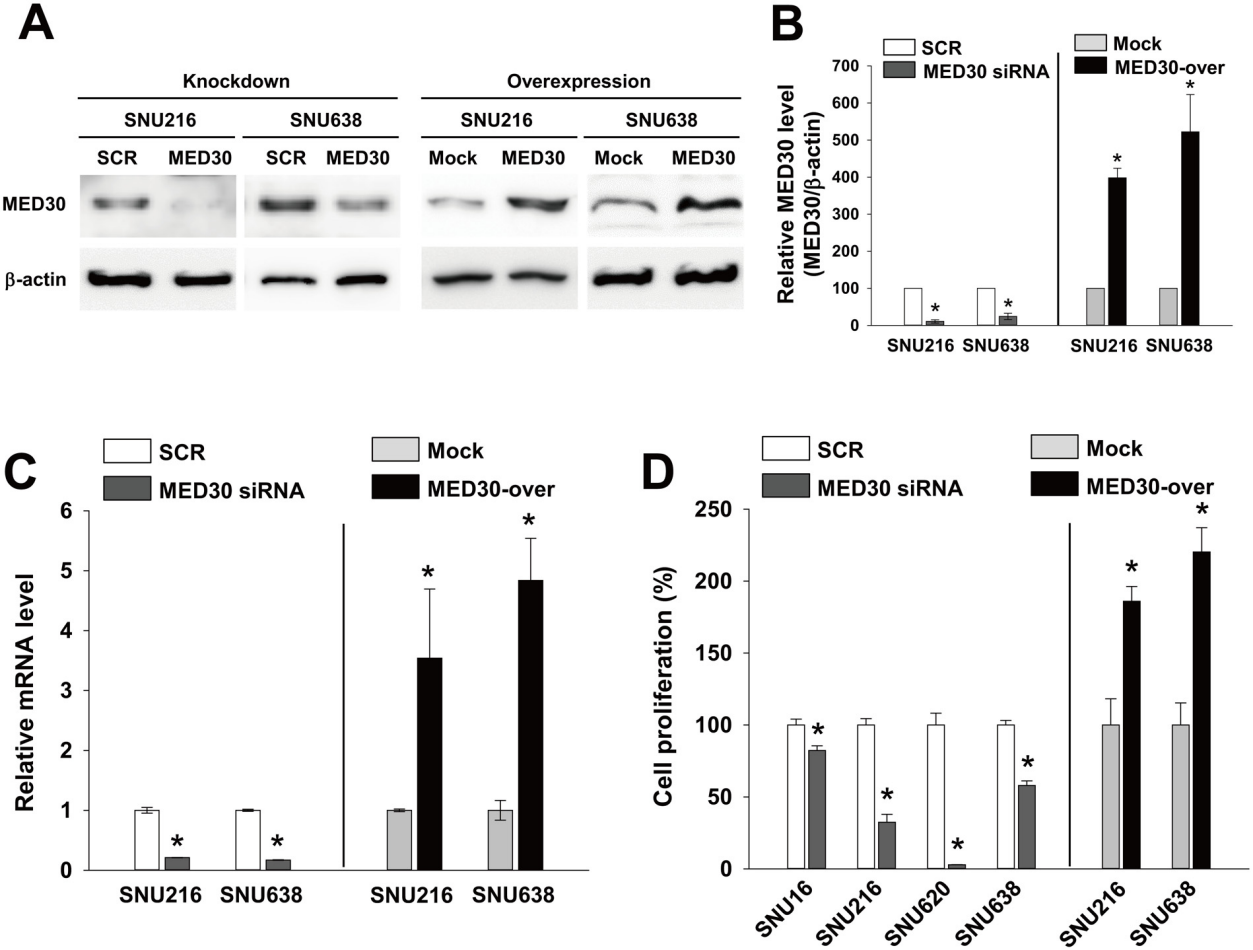
MED30 regulated the proliferation of gastric cancer cells. Gastric cancer cells (SNU216 and SNU638) were transfected with MED30 siRNA (100 nM) or scrambled (SCR) siRNA. MED30 knockdown efficiency was determined by western blot (A) and real-time PCR (C) at 48 hr post-transfection. MED30 expression was also examined in cells stably overexpressing MED30 (MED30-over) and in empty control vector-transfected (Mock) cells. (B) MED30 protein levels relative to β-actin were quantified using Multi Gauge V3.1 software. Values indicate MED30 to β-actin band intensity ratios and are the means ± SDs of three independent experiments performed in triplicate. *, *p* < 0.01 (Student’s *t* test, versus SCR or Mock). (D) Effect of MED30 knockdown or overexpression on the proliferation of gastric cancer cells (SNU16, SNU216, SNU620, and SNU638). To check the effect of MED30 knockdown, five days after the transfection with 100 nM MED30 siRNA or SCR siRNA, cell viability assays were performed, and to examine the effect of MED30 overexpression, cell viability assays were performed after 3 days of incubation. Values are the means ± SDs of three independent experiments performed in quadruplicate. *, *p*< 0.01 (Student’s *t* test, versus SCR or Mock).

To examine roles of MED30 in gastric carcinogenesis, we examined the effects of MED30 knockdown or overexpression on the proliferation, migration, and invasion of the six gastric cancer cell lines (SNU1, SNUI16, SNU216, SNU620, SNU638, and NCI-N87 cells). Real-time PCR and western blot were used to analyze the knockdown and overexpression of MED30 in SNU216 and SNU638 cells (Fig 2A–2C). When these cells were transfected with MED30 siRNA (100 nM), MED30 expression diminished at the protein and mRNA levels by about 80% two days later versus SCR siRNA, and MED30 overexpression by cDNA transfection increased MED30 mRNA and protein levels versus empty control vector-transfected cells (mock cells) by more than 3-fold.

We next investigated the role of MED30 in the proliferation of cancer cells. Proliferation assays were performed 5 days after transfection with SCR or MED30 siRNA. Knockdown of MED30 inhibited the proliferations of all gastric cancer cells tested (SNU16, SNU216, SNU620, and SNU638) versus SCR by 18%, 68%, 98%, and 42%, respectively (Fig 2D). Similar results were observed when we performed proliferation assays two or three days after transfection (data not shown). Consistently, MED30 overexpression enhanced the growths of SNU216 and SNU638 cells by 1.9 and 2.2 fold, respectively, versus mock cells.

**Fig 3:**
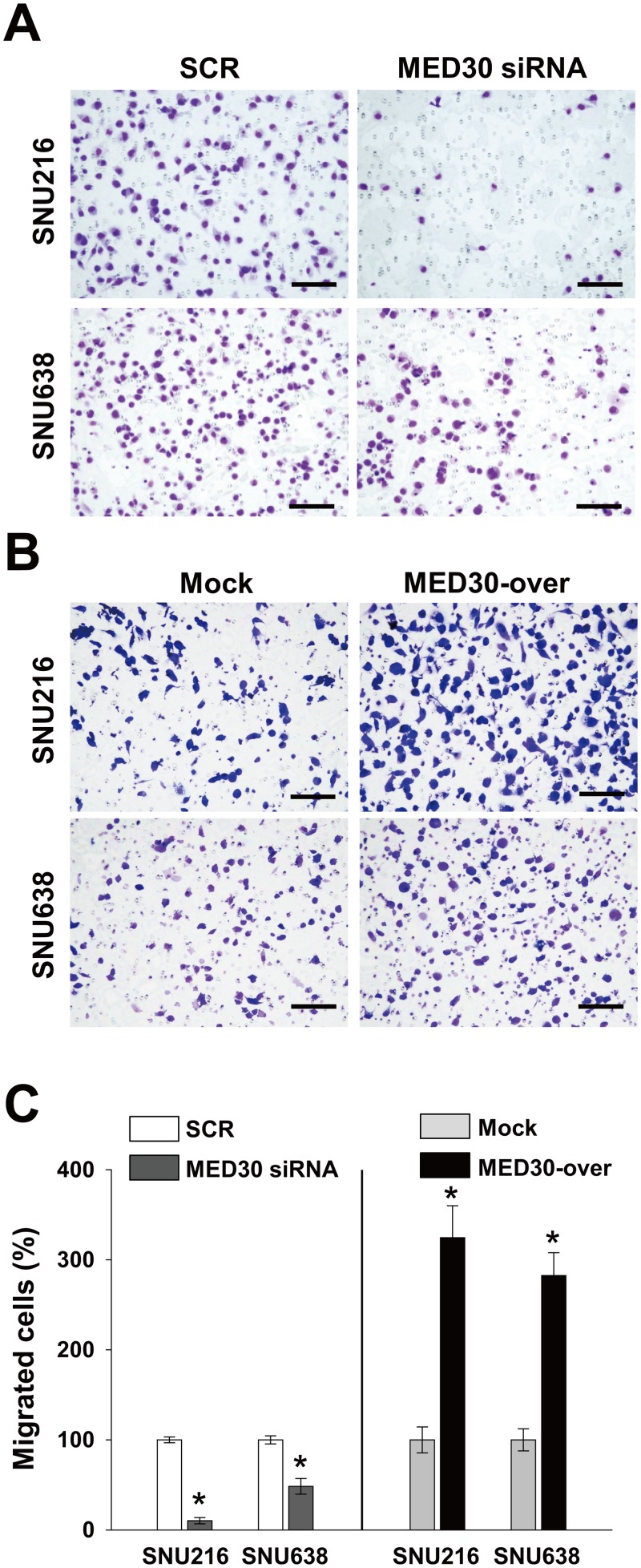
MED30 accelerated the migration of gastric cancer cells. Cell migration was evaluated using a Boyden chamber assay as described in ‘Materials and Methods’. (A) MED30 knockdown with MED30 siRNA significantly inhibited the FBS-induced migration of SNU216 and SNU638 cells compared with SCR siRNA. (B) MED30 overexpression (MED30-over) significantly increased migration compared to the mock control. (C) Migrated cells were counted and results are presented as a bar graph. Values are the means ± SDs of three independent experiments performed in triplicate. *, *p*< 0.01 (Student’s *t* test, versus SCR or Mock). Bar; 100 μm.

**Fig 4:**
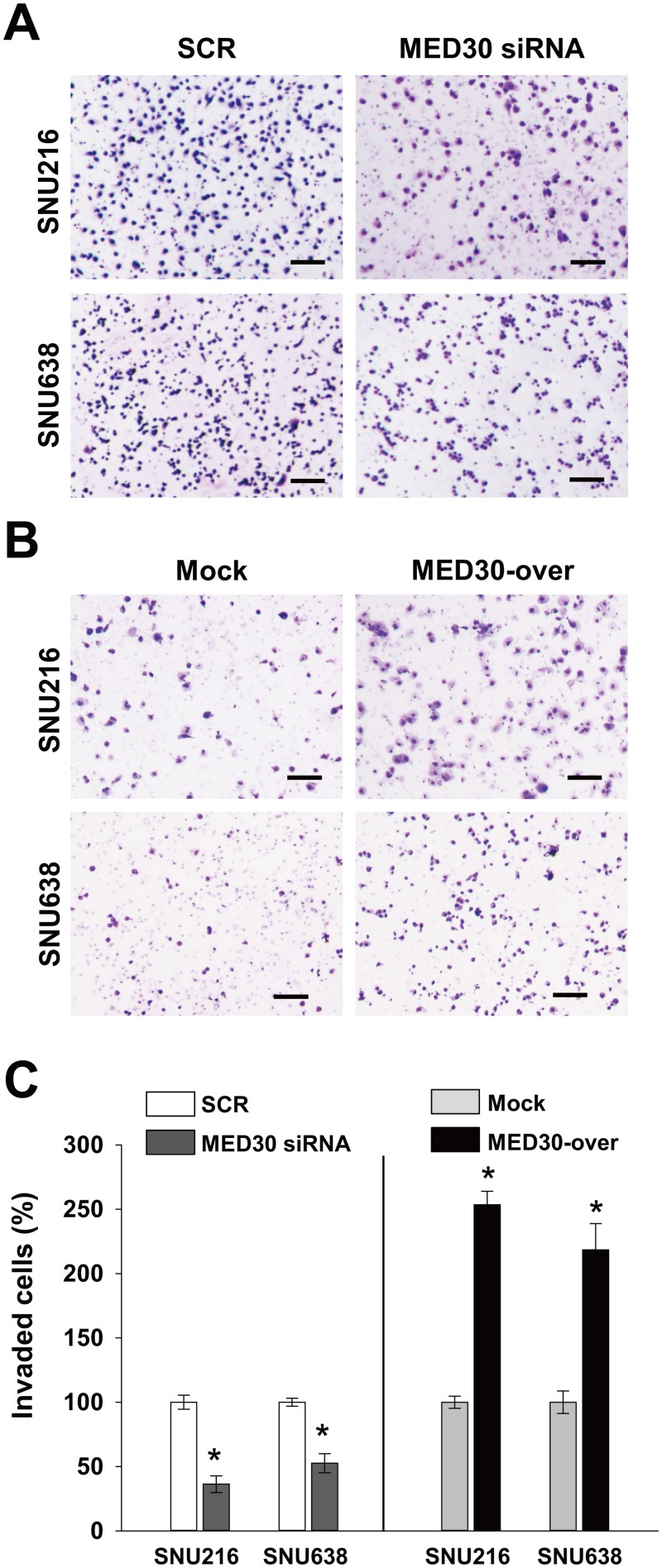
MED30 induced the invasion of gastric cancer cells. Cell invasion was examined using a Matrigel invasion assay. (A) Knockdown of MED30 significantly inhibited the FBS-induced invasions of SNU216 and SNU638 cells as compared with SCR siRNA. (B) MED30 overexpression (MED30-over) enhanced cell invasion compared with the mock control. (C) Invaded cells were counted and results are presented as a bar graph. Values are the means ± SDs of three independent experiments performed in triplicate. *, *p*< 0.01 (Student’s *t* test, versus SCR or Mock). Bar; 100 μm.

To determine the role played by MED30 in the migration of gastric cancer cells, we used a Boyden chamber assay. Knockdown of MED30 decreased FBS-induced migrations of SNU216 and SNU638 cells by 90% and 52%, respectively, versus SCR transfected cells (Fig 3A and 3C). Moreover, MED30 overexpression increased the FBS-induced migration of SNU216 and SNU638 cells by 3.2 and 2.8 fold, respectively, versus mock cells (Fig 3B and 3C). These results led us to examine the role of MED30 in the invasion of gastric cancer cells. In a Matrigel invasion assay, MED30 siRNA inhibited FBS-induced invasions of SNU216 and SNU638 cells versus SCR siRNA by 64% and 47%, respectively (Fig 4A and 4C), and MED30 overexpression accelerated the FBS-induced invasions of SNU216 and SNU638 cells versus mock cells by 2.5 and 2.2 fold, respectively (Fig 4B and 4C).

### Effect of MED30 knockdown on *in vivo* tumorigenicity

**Fig 5:**
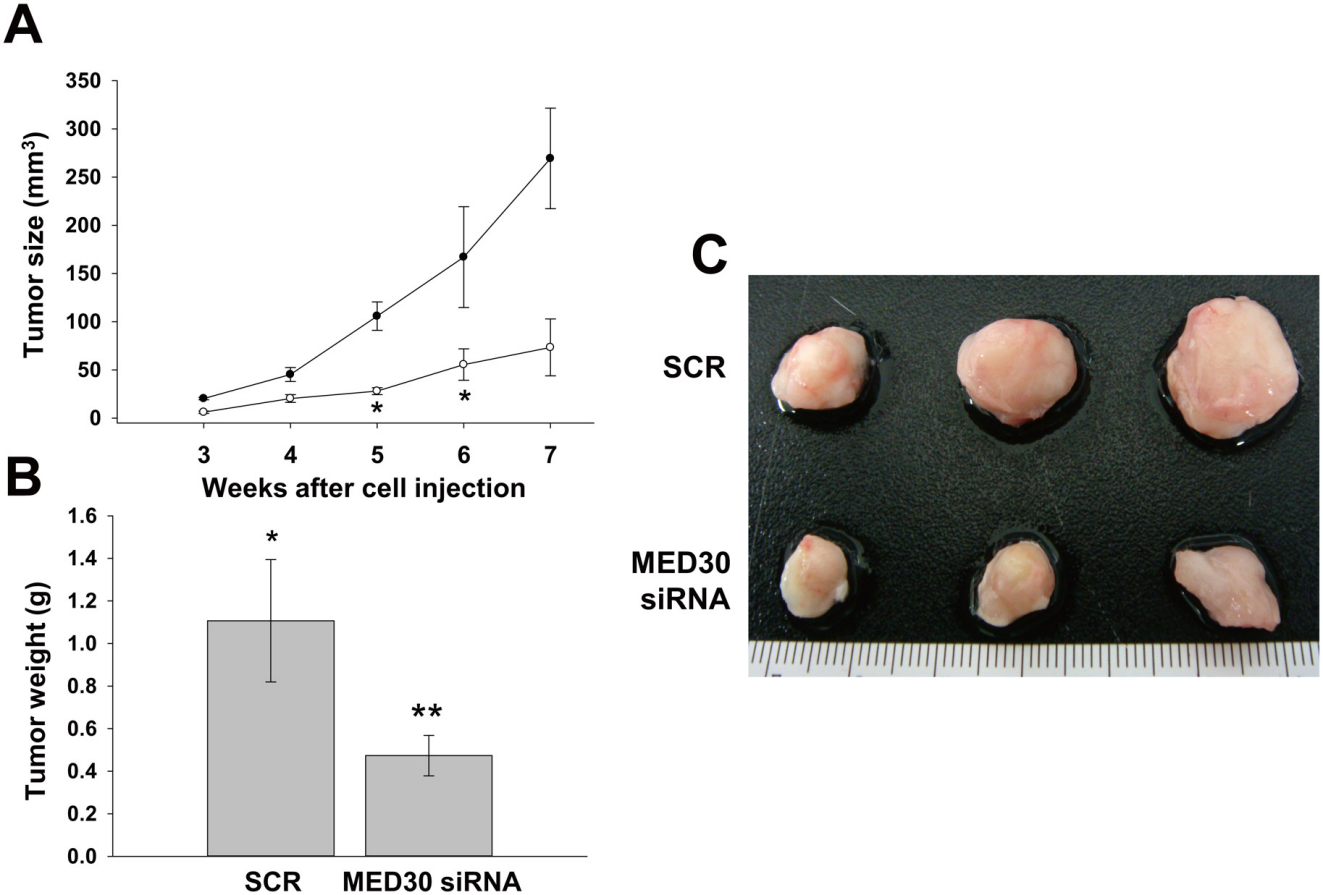
Knockdown of MED30 suppressed tumor growth in SCID mice. (A) SNU638 cells were transfected with MED30 or SCR siRNA and then injected subcutaneously into two sites per mouse. Tumor volumes were measured weekly from week three to seven post-injection. At week seven, mice were sacrificed and tumor volumes (B) and weights (C) were measured. Values are the means ± SDs of three independent experiments performed in triplicate. *, *p*< 0.01, **p< 0.05 (Student’s *t* test or one way ANOVA, versus SCR or Mock cells).

To examine the effect of MED30 on tumor growth, we subcutaneously injected SNU638 cells transfected with SCR or MED30 siRNA into SCID mice, and then monitored tumor growth for seven weeks (Fig 5A). Palpable tumors were detected within three weeks in the SCR control sides. However, cancer cells transfected with MED30 siRNA exhibited slower growth. Seven weeks after injection, mice were sacrificed, and tumor volumes and weights were measured (Fig 5B and 5C). The results showed MED30 knockdown significantly reduced tumor volumes and weights.

### Regulation of EMT pathway by MED30

**Fig 6:**
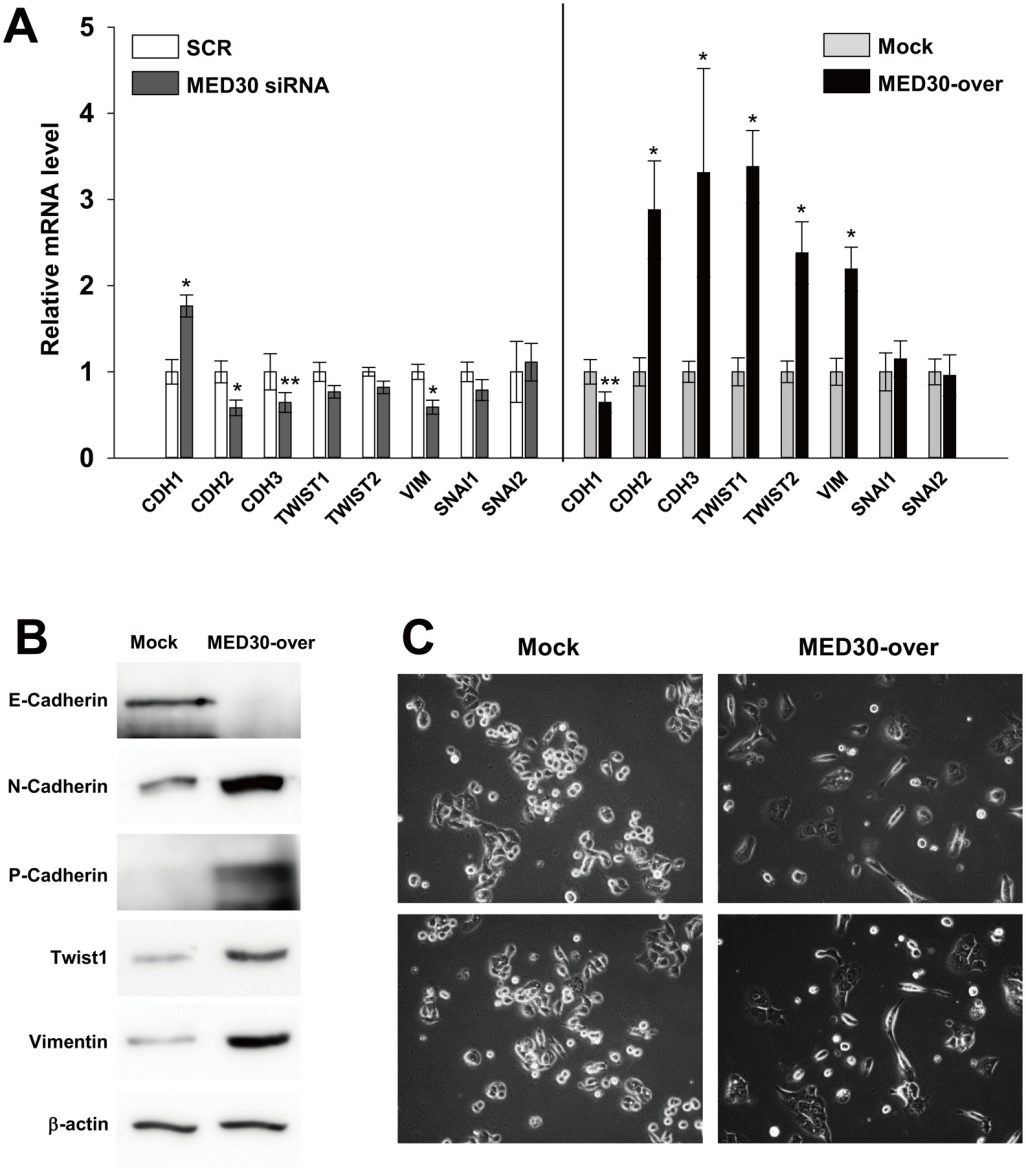
MED30 induced EMT in gastric cancer cells. Real-time PCR (A) and western blot analysis (B) of E-cadherin (CDH1), N-cadherin (CDH2), P-cadherin (CDH3), twist family bHLH transcription factor 1 (TWIST1/2), vimentin (VIM), and snail family zinc finger 1 and 2 (SNAI1/2) were performed after the knockdown or overexpression of MED30 in SNU638 cells. GAPDH and β-actin were used as internal control for real-time PCR and western blot analysis respectively. Values are the means ± SDs of three independent experiments performed in quadruplicate. *, *p*< 0.01, **p< 0.05 (Student’s *t* test, versus Mock). (C) SNU638 cells (MED30-over and mock) were grown in medium containing 10% FBS. After 2 days of culture, the morphologies of mock and MED30-overexpressing cells were observed by bright field microscopy.

To determine the mechanism underlying the promotion of gastric carcinogenesis by MED30, we investigated the expression patterns of genes involved in epithelial-mesenchymal transition (EMT: a key process in metastasis and invasion) by real-time PCR. Knockdown of MED30 slightly increased the level of E-cadherin (CDH1) mRNA in SNU638 cells versus SCR transfection, but decreased expressions of N-cadherin (CDH2), P-cadherin (CDH3) and vimentin (VIM) by 42%, 36%, and 41%, respectively (Fig 6A). Consistently, MED30 overexpression decreased CDH1 mRNA levels by 35% versus mock cells, but increased the expressions of N-cadherin (CDH2), P-cadherin (CDH3), twist family bHLH transcription factor 1 and 2 (TWIST1/2), and vimentin (VIM) by 2.9, 3.3, 3.4, 2.4, 2.2, and 4.2 fold, respectively (Fig 6A). The mRNA levels of snail family zinc finger 1 and 2 (SNAI1/2) were unchanged. We then confirmed that MED30 overexpression also increased the protein levels of E-cadherin, N-cadherin, P-cadherin, Twist1, and vimentin (Fig 6B). An examination of the morphology of SNU638 cells revealed that MED30 overexpression triggered a transition from a cobblestone-like to an elongated fibroblast-like morphology (Fig 6C), whereas MED30 knockdown did not alter morphology (data not shown). These results suggest that MED30 positively regulates EMT.

## Discussion

The functional roles of the MED subunit MED30 (also known as TRAP25) are poorly understood. In a recent report, MED30 was found to play a significant role in regulating mitochondrial functions and integrity in homozygous mice [19]. In the present study, we examined the functional roles of MED30 during gastric cancer progression. It was found MED30 regulated the proliferation, migration, and invasion of gastric cancer cells and their *in vivo* tumorigenicity. After ensuring knockdown and overexpression efficiency by quantitative real-time PCR and western blot analysis (Fig 2), we found that MED30 knockdown remarkably suppressed the proliferation, migration, and invasion of gastric cancer cells, and that MED30 overexpression had the opposite effects (Figs 2–4). Moreover MED30 knockdown reduced *in vivo* tumorigenicity (Fig 5). Furthermore, we also found that MED30 was overexpressed in gastric cancer tissues and gastric cancer cell lines (Fig 1). These results suggest that MED30 might play important pathophysiological roles during gastric carcinogenesis.

Since specific MED subunits are associated with signal-activated transcription factors and RNA polymerase II (Pol II) [20] and function as conduits and integrators for channeling different signaling pathways, such as, the nuclear hormone receptor pathway (via MED1) [21], the TGF-β-signaling pathway (via MED12 or MED15) [22, 23], the Wnt-signaling pathway (via MED12) [24], and the Ras-MAPK signaling pathway (via MED23) [25–27]. It was proposed that these signaling pathways can induce EMT [28–32], which is known to be associated with the metastasis, invasion, proliferation, and chemoresistance of epithelial cancer [29, 33, 34]. Therefore, we asked whether MED30 triggers EMT. MED30 induced the expressions of EMT-related genes (N-cadherin; CDH2, P-cadherin; CDH3, twist related protein 1 and 2; TWIST1/2, vimentin; VIM), but inhibited the expression of E-cadherin (CDH1) a known tumor suppressor (Fig 6A and 6B). Furthermore, MED30 overexpression induced a fibroblast-like morphology (Fig 6C), which suggests MED30 triggers EMT in gastric cancer cells.

Summarizing, the present study supports the notion that MED30 acts as an oncogene during gastric carcinogenesis and suggests MED30 might regulate EMT in gastric cancer. Although further studies are required to determine how MED30 triggers EMT, our results suggest that MED30 be considered a novel molecular target for the treatment of gastric cancer.

## References

[1] Smith MG, Hold GL, Tahara E, El-Omar EM. Cellular and molecular aspects of gastric cancer. World journal of gastroenterology: WJG. 2006;12(19):2979–90. Epub 2006/05/24. . 16718776 10.3748/wjg.v12.i19.2979 PMC4124370

[2] Jemal A, Bray F, Center MM, Ferlay J, Ward E, Forman D. Global cancer statistics. CA: a cancer journal for clinicians. 2011;61(2):69–90. Epub 2011/02/08. 10.3322/caac.20107 . 21296855

[3] Crew KD, Neugut AI. Epidemiology of gastric cancer. World journal of gastroenterology: WJG. 2006;12(3):354–62. Epub 2006/02/21. 16489633 10.3748/wjg.v12.i3.354 PMC4066052

[4] Lauren P. The Two Histological Main Types of Gastric Carcinoma: Diffuse and So-Called Intestinal-Type Carcinoma. An Attempt at a Histo-Clinical Classification. Acta pathologica et microbiologica Scandinavica. 1965;64:31–49. Epub 1965/01/01. . 14320675 10.1111/apm.1965.64.1.31

[5] Pacelli F, Papa V, Caprino P, Sgadari A, Bossola M, Doglietto GB. Proximal compared with distal gastric cancer: multivariate analysis of prognostic factors. The American surgeon. 2001;67(7):697–703. Epub 2001/07/14. . 11450793

[6] Tay ST, Leong SH, Yu K, Aggarwal A, Tan SY, Lee CH, et al A combined comparative genomic hybridization and expression microarray analysis of gastric cancer reveals novel molecular subtypes. Cancer research. 2003;63(12):3309–16. Epub 2003/06/18. . 12810664

[7] Ooi CH, Ivanova T, Wu J, Lee M, Tan IB, Tao J, et al Oncogenic pathway combinations predict clinical prognosis in gastric cancer. PLoS genetics. 2009;5(10):e1000676 Epub 2009/10/03. 10.1371/journal.pgen.1000676 19798449 PMC2748685

[8] Shah MA, Khanin R, Tang L, Janjigian YY, Klimstra DS, Gerdes H, et al Molecular classification of gastric cancer: a new paradigm. Clinical cancer research: an official journal of the American Association for Cancer Research. 2011;17(9):2693–701. Epub 2011/03/25. 10.1158/1078-0432.CCR-10-2203 21430069 PMC3100216

[9] Tan IB, Ivanova T, Lim KH, Ong CW, Deng N, Lee J, et al Intrinsic subtypes of gastric cancer, based on gene expression pattern, predict survival and respond differently to chemotherapy. Gastroenterology. 2011;141(2):476–85, 85 e1–11. Epub 2011/06/21. 10.1053/j.gastro.2011.04.042 21684283 PMC3152688

[10] Deng N, Goh LK, Wang H, Das K, Tao J, Tan IB, et al A comprehensive survey of genomic alterations in gastric cancer reveals systematic patterns of molecular exclusivity and co-occurrence among distinct therapeutic targets. Gut. 2012;61(5):673–84. Epub 2012/02/09. 10.1136/gutjnl-2011-301839 22315472 PMC3322587

[11] Baek HJ, Malik S, Qin J, Roeder RG. Requirement of TRAP/mediator for both activator-independent and activator-dependent transcription in conjunction with TFIID-associated TAF(II)s. Molecular and cellular biology. 2002;22(8):2842–52. Epub 2002/03/23. 11909976 10.1128/MCB.22.8.2842-2852.2002 PMC133729

[12] Kornberg RD. Mediator and the mechanism of transcriptional activation. Trends in biochemical sciences. 2005;30(5):235–9. Epub 2005/05/18. 10.1016/j.tibs.2005.03.011 . 15896740

[13] Malik S, Roeder RG. Dynamic regulation of pol II transcription by the mammalian Mediator complex. Trends in biochemical sciences. 2005;30(5):256–63. Epub 2005/05/18. 10.1016/j.tibs.2005.03.009 . 15896744

[14] Balamotis MA, Pennella MA, Stevens JL, Wasylyk B, Belmont AS, Berk AJ. Complexity in transcription control at the activation domain-mediator interface. Science signaling. 2009;2(69):ra20 Epub 2009/05/07. 10.1126/scisignal.1164302 19417216 PMC2774526

[15] Taatjes DJ. The human Mediator complex: a versatile, genome-wide regulator of transcription. Trends in biochemical sciences. 2010;35(6):315–22. Epub 2010/03/20. 10.1016/j.tibs.2010.02.004 20299225 PMC2891401

[16] Yin JW, Wang G. The Mediator complex: a master coordinator of transcription and cell lineage development. Development. 2014;141(5):977–87. Epub 2014/02/20. 10.1242/dev.098392 . 24550107

[17] Schiano C, Casamassimi A, Rienzo M, de Nigris F, Sommese L, Napoli C. Involvement of Mediator complex in malignancy. Biochimica et biophysica acta. 2014;1845(1):66–83. Epub 2013/12/18. 10.1016/j.bbcan.2013.12.001 . 24342527

[18] Ding XF, Huang GM, Shi Y, Li JA, Fang XD. Med19 promotes gastric cancer progression and cellular growth. Gene. 2012;504(2):262–7. Epub 2012/05/09. 10.1016/j.gene.2012.04.033 . 22565189

[19] Krebs P, Fan W, Chen YH, Tobita K, Downes MR, Wood MR, et al Lethal mitochondrial cardiomyopathy in a hypomorphic Med30 mouse mutant is ameliorated by ketogenic diet. Proceedings of the National Academy of Sciences of the United States of America. 2011;108(49):19678–82. Epub 2011/11/23. 10.1073/pnas.1117835108 22106289 PMC3241770

[20] Woychik NA, Hampsey M. The RNA polymerase II machinery: structure illuminates function. Cell. 2002;108(4):453–63. Epub 2002/03/23. . 11909517 10.1016/s0092-8674(02)00646-3

[21] Kang YK, Guermah M, Yuan CX, Roeder RG. The TRAP/Mediator coactivator complex interacts directly with estrogen receptors alpha and beta through the TRAP220 subunit and directly enhances estrogen receptor function in vitro. Proceedings of the National Academy of Sciences of the United States of America. 2002;99(5):2642–7. Epub 2002/02/28. 10.1073/pnas.261715899 11867769 PMC122401

[22] Kato Y, Habas R, Katsuyama Y, Naar AM, He X. A component of the ARC/Mediator complex required for TGF beta/Nodal signalling. Nature. 2002;418(6898):641–6. Epub 2002/08/09. 10.1038/nature00969 . 12167862

[23] Huang S, Holzel M, Knijnenburg T, Schlicker A, Roepman P, McDermott U, et al MED12 controls the response to multiple cancer drugs through regulation of TGF-beta receptor signaling. Cell. 2012;151(5):937–50. Epub 2012/11/28. 10.1016/j.cell.2012.10.035 23178117 PMC3672971

[24] Kim S, Xu X, Hecht A, Boyer TG. Mediator is a transducer of Wnt/beta-catenin signaling. The Journal of biological chemistry. 2006;281(20):14066–75. Epub 2006/03/28. 10.1074/jbc.M602696200 . 16565090

[25] Wang G, Balamotis MA, Stevens JL, Yamaguchi Y, Handa H, Berk AJ. Mediator requirement for both recruitment and postrecruitment steps in transcription initiation. Molecular cell. 2005;17(5):683–94. Epub 2005/03/08. 10.1016/j.molcel.2005.02.010 . 15749018

[26] Yang X, Zhao M, Xia M, Liu Y, Yan J, Ji H, et al Selective requirement for Mediator MED23 in Ras-active lung cancer. Proceedings of the National Academy of Sciences of the United States of America. 2012;109(41):E2813–22. Epub 2012/09/19. 10.1073/pnas.1204311109 22988093 PMC3478617

[27] Stevens JL, Cantin GT, Wang G, Shevchenko A, Berk AJ. Transcription control by E1A and MAP kinase pathway via Sur2 mediator subunit. Science. 2002;296(5568):755–8. Epub 2002/04/06. 10.1126/science.1068943 . 11934987

[28] Xie M, Zhang L, He CS, Xu F, Liu JL, Hu ZH, et al Activation of Notch-1 enhances epithelial-mesenchymal transition in gefitinib-acquired resistant lung cancer cells. Journal of cellular biochemistry. 2012;113(5):1501–13. Epub 2011/12/17. 10.1002/jcb.24019 . 22173954

[29] Espinoza I, Miele L. Deadly crosstalk: Notch signaling at the intersection of EMT and cancer stem cells. Cancer letters. 2013;341(1):41–5. Epub 2013/08/27. 10.1016/j.canlet.2013.08.027 . 23973264

[30] Zavadil J, Cermak L, Soto-Nieves N, Bottinger EP. Integration of TGF-beta/Smad and Jagged1/Notch signalling in epithelial-to-mesenchymal transition. The EMBO journal. 2004;23(5):1155–65. Epub 2004/02/21. 10.1038/sj.emboj.7600069 14976548 PMC380966

[31] Kim H, Choi JA, Kim JH. Ras Promotes Transforming Growth Factor-beta (TGF-beta)-induced Epithelial-Mesenchymal Transition via a Leukotriene B4 Receptor-2-linked Cascade in Mammary Epithelial Cells. The Journal of biological chemistry. 2014;289(32):22151–60. Epub 2014/07/06. 10.1074/jbc.M114.556126 24990945 PMC4139228

[32] Yu C, Liu Y, Huang D, Dai Y, Cai G, Sun J, et al TGF-beta1 mediates epithelial to mesenchymal transition via the TGF-beta/Smad pathway in squamous cell carcinoma of the head and neck. Oncology reports. 2011;25(6):1581–7. Epub 2011/04/12. 10.3892/or.2011.1251 . 21479366

[33] Cano A, Perez-Moreno MA, Rodrigo I, Locascio A, Blanco MJ, del Barrio MG, et al The transcription factor snail controls epithelial-mesenchymal transitions by repressing E-cadherin expression. Nature cell biology. 2000;2(2):76–83. Epub 2000/02/03. 10.1038/35000025 . 10655586

[34] Yan S, Wang Y, Yang Q, Li X, Kong X, Zhang N, et al Low-dose radiation-induced epithelial-mesenchymal transition through NF-kappaB in cervical cancer cells. International journal of oncology. 2013;42(5):1801–6. Epub 2013/03/14. 10.3892/ijo.2013.1852 . 23483258

